# Use of magnetic resonance image‐guided radiotherapy for breast cancer: a scoping review

**DOI:** 10.1002/jmrs.545

**Published:** 2021-09-15

**Authors:** Alexandra Berlangieri, Sarah Elliott, Jason Wasiak, Michael Chao, Farshad Foroudi

**Affiliations:** ^1^ Olivia Newton John Cancer Wellness and Research Centre (ONCWRC) Austin Health Heidelberg Victoria Australia

**Keywords:** Accelerated partial breast irradiation, breast cancer, electron return effect, MR simulation, MRI guided radiotherapy, MR‐linac

## Abstract

In recent years, we have seen the integration of magnetic resonance imaging (MRI) simulators into radiotherapy centres and the emergence MR linear accelerators (MR‐linac). Currently, there are limited studies to demonstrate the clinical effectiveness of MRI guided radiotherapy (MRIgRT) treatment for breast cancer patients. The objective of this scoping review was to identify and map the existing evidence surrounding the clinical implementation of MRIgRT for breast cancer patients. We also identified the challenges and knowledge gaps in the literature. The scoping review was reported in accordance with the Preferred Reporting Items for Systematic reviews and Meta‐Analysis (PRISMA) extension for Scoping Reviews reporting guidelines. Titles and abstracts were screened by two independent reviewers. Quantitative and qualitative data were extracted and summarised using thematically organised tables. Results identify that accelerated partial breast irradiation (APBI) is the most common form of treatment for MRIgRT. The presence of the magnet does not affect target coverage or violate organ at risk (OAR) constraints compared to standard radiotherapy methods. Consideration is advised for skin and chest wall (CW) due to the electron return effect (ERE) and areas such as armpit and chin due to the electron stream effect (ESE). Clinically, bolus has been used to protect and prevent unwanted dose in these areas. Overall treatment for APBI on the MR‐linac is feasible.

## Graphical abstract

Accelerated partial breast irradiation (APBI) is the most common form of treatment for MRIgRT. The presence of the magnet does not affect target coverage or violate organ at risk (OAR) constraints compared to standard RT methods. Consideration is advised for skin and chestwall (CW) due to the electron return effect (ERE) and areas such as armpit and chin due to the electron stream effect (ESE).

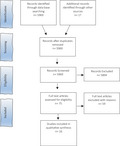

## Introduction

The development of magnetic resonance imaging‐guided radiation therapy (MRIgRT) has greatly improved imaging visibility in the radiation therapy (RT) domain. Magnetic resonance imaging (MRI) uses a strong magnetic field to provide superior soft tissue delineation and an increased sensitivity for tumour detection compared to CBCT.^1^ In 2008, an MR‐linac prototype was developed at University Medical Centre Utrecht (UMC)^2^ combining a Philips (Best, The Netherlands) 1.5 T MRI scanner with an Elekta AB (Stockholm, Sweden) linear accelerator (linac). The system has since evolved into the Elekta Unity system.^3^ The MRIdian (ViewRay Inc., Mountain View, CA, USA) is an alternative system, initially designed with three ^60^Co sources 120 degrees apart and equipped with a 0.35 T static MRI system.^4^ A more recent generation has replaced this system, now with a 6 MV flattening filter free linac and a 0.35 T superconducting magnet.^5^ These machines acquire intrafractional images with increased target and organ at risk (OAR) visibility, making online planning adaptations possible and enabling a more personalised treatment approach for patients.^6^ To improve the integration of MR‐linacs within departments, MR simulators are being used for preliminary planning scans. These machines include flat table tops, localisation for stabilisation equipment and external localising lasers.^7^ Imaging on the MR simulator or MR‐linac do not contribute any extra dose to the patient compared to CBCT, which is desirable when considering the ‘as low as reasonably achievable’ (ALARA) principle. Superior image quality in MR simulation and treatment promotes a reduction in treatment margins. Reducing margins limits dose delivered to OAR. This can reduce the chances of adverse effects, increase quality of life and create greater patient outcomes.^8^ Owing to these benefits, fractional dose can be escalated with fewer fractionations.^9^ These factors can alleviate appointment burden for patients and also lead to improved locoregional control.

In 2018, breast cancer was the fifth leading cause of cancer death in Australia and also the second most common cause of death from cancer amongst females.^10^ In 2021, it has been predicted that breast cancer will be the most commonly diagnosed cancer. The majority of female breast cancers in 2011 were diagnosed as Stage 1–2 (77%).^11^ Early‐stage breast cancers are a strong candidate for this novel technology. The tumour or tumour cavity is highly visible on MR imaging making localisation straightforward, removing the need to treat the whole breast.^12^ This form of treatment is known as accelerated partial breast irradiation (APBI) and is commonly used in brachytherapy.^13^ However, as departments begin to have access to the MR‐linac/simulator, there is a need to examine the use of the novel technology in simulation and image guided treatment for breast cancer patients.

The aim of the scoping review was to summarise current literature, with an emphasis on summarising the patient pathway of simulation, planning and treatment for early‐stage breast cancer. In addition, considerations and knowledge gaps were evaluated to assist departments in the use of the MRL with breast cancer patients.

## Materials and Methods

This review followed the five stages outlined in the Arskey and O’Malley^14^ framework, which has been further developed by Levac et al.^15^ Comprehensive details regarding the current paper can be read in the authors scoping review protocol.^16^

### Stage 1: Identifying research question

The aim was to scope the existing literature to identify the evidence, map‐existing literature and define the optimal use of the MR simulator and MR‐linac in breast radiotherapy. Therefore, the research question was derived from the broad scope that characterises a scoping review. What is the range of existing evidence surrounding the clinical implementation of MRIgRT in patients with breast cancer?

### Stage 2: Identifying relevant studies

A systematic literature search was conducted to identify intervention studies that reported on an MR‐linac and breast RT. The electronic search strategy included keywords such as ‘breast cancer’, ‘magnetic resonance imaging’, ‘MR‐linac’, ‘image guidance’ and their derivates using MEDLINE (Ovid) and EMBASE (Ovid) library data bases (January 2010–December 2020). Grey literature sources searched included clinical trials using the World Health Organisation (WHO) International Clinical Trials Registry Platform. Key journals and conference papers were also screened. We included systematic reviews, randomised and non‐randomised controlled studies published in English. Literature assessed was required to examine the use of MRIgRT in adults with breast cancer, regardless of cancer stage or severity. Refer to Table 1 for study inclusion and exclusion criteria.

**Table 1:** Study inclusion and exclusion criteria.

Study characteristics	Inclusion criteria	Exclusion criteria
Design	Systematic review Randomised and non‐randomised Controlled studies Clinical Trials	Case report/study Descriptive report
Publication	Peer reviewed journal Published in English Abstract and full text available 2010 to December 2020	Doctoral thesis Conference proceeding, abstract or poster
Participants	Breast cancer only Adults ≥18 years Any tumour stage	Any cancer excluding breast metastases, nodal spread
Intervention	MR‐Linac MR Simulator Any type of radiation therapy intervention, for example, Brachytherapy, VMAT, IMRT, 3DCRT	Standard diagnostic MRI scans used in a radiation therapy setting

### Stage 3: Study selection

Two reviewers independently screened citation titles and abstracts, then reviewed potentially relevant articles in full. *Covidence* ^17^ was used for efficient screening. Any disagreement was resolved by a third author or group discussion and if consensus could not be achieved, a fourth review author was consulted for final study arbitration.

### Stage 4: Data charting process

A charting form in Microsoft Excel (Microsoft, Redmond) was used to categorise the studies according to their focus. Data analysis involved quantitative (e.g. frequencies) and qualitative (e.g. content and thematic analysis) methods. The following data items were extracted: general data (title, year of publication, authors name); methodological data (research design, setting, sample number, patient characteristics); machine data (beam energy, magnet strength), planning parameters, type of treatment and general patient outcomes.

### Stage 5: Summarising results

The results were organised under the following categories: patient characteristics, simulation, planning parameters, treatment parameters, APBI, online adaptive radiation therapy (ART), dose to OAR, electron return effect (ERE), electron stream effect (ESE) and machine geometry. We reported the review following the Preferred Reporting Items for Systematic Review and Meta‐Analysis (PRISMA) guidelines.^18^ This review has been registered with Open Science Framework and ORCID (https://orcid.org/0000‐0003‐0795‐1995).

## Results

**Figure 1:**
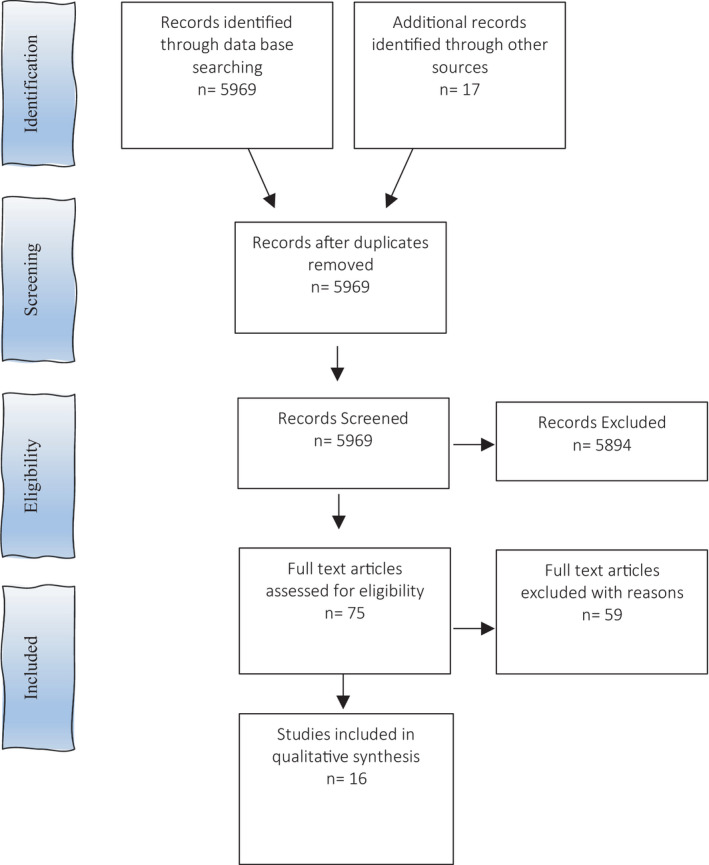
PRISMA diagram of selection of studies.

We identified 16 eligible studies based on the literature search strategy. The study selection process is outlined in Figure 1. The majority of these studies were retrospective (*n* = 10) from the Netherlands, Iran, Korea, USA, Canada and Germany.^19^, ^20^, ^21^, ^22^, ^23^, ^24^, ^25^, ^26^, ^27^ Two studies were prospective,^12^, ^28^ and the same number were longitudinal.^6^, ^9^ One position paper was included.^29^ The majority of studies were conducted on women, with two studies^30^, ^31^ performed on phantoms. Sample sizes ranged from 1 to 209. We mapped the distribution of studies according to the study design (Table 2). Studies were divided between MR planning dosimetry, MR treatment studies and one quality assurance study.^32^ Study themes were varied, the most common being APBI (*n* = 10). There was a significant deficiency in the number of articles addressing MR simulation and planning. Limited short‐term patient outcomes following MRIgRT were available; early results of a Phase 1 trial indicated zero failures and excellent‐to‐good cosmetic outcomes^9^ and a first‐in human publication reported grade 1 breast oedema at 3 months.^27^ The longest follow‐up time was 4.5 years.^9^

**Table 2:** General study characteristics and general findings of included studies.

Author	Country	Sample size	Study design	General findings
van Heijst 2013^19^	Netherlands	10	Retrospective	ERE can increase skin dose for WBI on the MRL. In APBI the induced effects of ERE on skin is negligible
Esmaeeli 2014^20^	Iran	4	Retrospective	A reversible magnetic field can reduce dose to the lung and heart, whilst also producing a sharp DVH for the PTV
Kim 2015^21^	Korea	11	Retrospective	In OAR analysis, a significant effect of the magnetic field was not observed with 0.35 T
Madhavi 2015^22^	Iran	2	Retrospective	When magnetic field is parallel to the photon beam axis, the radial spread of electrons is reduced resulting in dose reduction to the lungs
Acharya 2016^12^	USA	30	Prospective	Minimal intrafractional variation of the breast surgical cavity during APBI delivery and there is a good agreement between delivered and planned dose
Chen 2016^23^	USA	1	Retrospective	ERE can occur in the presence of a TMF. These changes can be substantially reduced, when the TMF is considered during IMRT/VMAT optimisation
Fischer‐Valuk 2017^6^	USA	82	Longitudinal	Breast was a common body site treated (26%) in first two and a half year experience. Candidates who are not suited for brachytherapy at Washington University Hospital are eligible for APBI
Kim 2017^24^	Canada	5	Retrospective	The magnetic field increases skin dose; however, this can be mitigated by increasing the number of beam angles
Jeon 2017^25^	Korea	37	Retrospective	Seromas exhibit exponential shrinkage during APBI. Frequent monitoring is essential for decision making regarding ART
Henke 2018^9^	USA	209	Longitudinal	In 4.5 year experience, MRIgRT used for hypofractionated APBI was a popular treatment. ART was advantageous for this type of treatment
Park 2018^28^	Germany	20	Prospective	Patients must be shielded from AES to avoid unwanted irradiation of skin outside the treatment field
Charaghvandi 2019^26^	Netherlands	20	Retrospective	Single‐dose APBI to the intact tumour is dosimetrically feasible. Prone position was advantageous for OAR dosimetry
An 2019^31^	Korea	Custom made phantom	Non‐patient controlled study	AES increased with the projected area of the cross‐section of the treatment beam. Shielding must be considered to prevent undesirable out‐of‐field irradiation
Nachbar 2020^27^	Germany	1	Retrospective	ESE is accurately calculated by the TPS and can be effectively reduced with a 1 cm bolus and is comparable to dose of CBCT based position verification. The additional ERE dose is not associated with increased risk of acute toxicity
Groot Koerkamp 2020^29^	Netherlands	–	Position paper	Treatment on an MRL can lead to margin reduction in the neoadjuvant and adjuvant PBI. Technical approaches and workflows are yet to be explicitly presented
Mönnich 2020^30^	Germany	106 clinical TPs (19 PBI)	Non‐patient controlled study	QA plans were measured on the Octavius phantom and the Octavius 1500MR chamber array with various positioning of the phantom on the MRL. PBI had a median gamma pass rate of 98.0%

AES, air electron stream; APBI, Accelerated partial breast irradiation; ART, adaptive radiation therapy; CBCT, cone beam computed tomography; DVH, dose volume histogram; ERE, electron return effect; IMRT, intensity modulated radiation therapy; MRIgRT, magnetic resonance image guided radiation therapy; MRL, magnetic resonance linear accelerator; OAR, organ at risk; PBI, partial breast irradiation; PTV, planning target volume; QA, quality assurance; T, Tesla; TMF, transverse magnetic field; TPS, treatment planning system; VMAT, volumetric modulated radiation therapy; WBI, whole breast irradiation.

### Patient selection

Patient age was reported in five studies, with all analyses on women over 39 years old. Laterality was described in eight studies; in total, there were 30 right‐ and 39 left‐sided tumours. No nodal involvement was reported in any of the studies. Two APBI studies were performed on preoperative patients.^19^, ^26^ Appropriate patients for APBI were early stage (Stage I–II) with intact tumours <3.0 cm or postoperative with negative surgical margins.^6^, ^9^, ^12^, ^19^, ^25^, ^27^, ^28^ Patients suitable for MRIgRT APBI were those unable to undergo high dose rate brachytherapy.^6^

### Simulation

CT simulation for breast radiotherapy is standard‐of‐care. In eight studies, radiotherapy simulation images were performed on both CT and MR. Of these studies, MRI imaging was performed on a diagnostic MRI (*n* = 2),^19^, ^26^ a dedicated radiotherapy MRI simulator (*n* = 3)^6^, ^9^, ^26^ or on the IGRT system (*n* = 4).^12^, ^25^, ^27^, ^28^ MR sequence details were only discussed in the position paper. The use of T1 without fat suppression is superior imaging for surgical clips. T1 with fat suppression (e.g. Dixon) can assist with differentiation between glandular breast and seroma. Whilst T2 with fat suppression (e.g. short inversion time recovery (STIR)) can be used for the visualisation of lumpectomy cavity and associated seroma and for discrimination between glandular breast tissue and tumour bed.^29^

The majority of studies simulated patients in the supine position with both arms up or the ipsilateral arm raised above the head. Patients were inclined on breast boards (Thorawedge^®^/Macromedics^®^). Jeon et al.^25^ simulated patients in supine position using a custom vacuum lockbag for arm elevation plus knee support. One study positioned a subgroup of patients in the prone position using a CDR prone breast board (Procline™).^23^

Two papers described the use of a dummy coil for CT simulation acquisition. This ensured coil attenuation was included during plan optimisation in the treatment planning system.^12^, ^28^ Groot Koerkamp et al.^29^ noted anterior coil placement could lead to deformation of the breast. MRI coils should avoid direct contact with the patient, preventing physical deformation such that the exact patient position between simulation and treatment can be produced for accurate dose delivery.^12^ Attentiveness is required when the prone position is used. Enough room is required to place a receiver coil on the back of the patient whilst ensuring the breast hangs freely without touching the MR table top.^29^ Assessment of target motion on a cine MRI in the sagittal plane was conducted during the simulation stage in one report.^6^ Simulation time is estimated to be 1.5 h.^6^

### Planning

Image fusion methods of MR to CT images were rigid (*n* = 2)^12^, ^27^ or deformable (*n* = 1).^28^ Contouring methods (i.e. manual versus auto‐segmentation) were not reported in detail. Clinical target volume (CTV) and planning target volume (PTV) contouring differed between the studies. Conservative studies applied an isotropic margin of 10 mm around the gross tumour volume (GTV) to form the PTV. Due to greater soft tissue contrast with daily MR treatment imaging, several utilised a decreased margin of CTV + 5 mm (*n* = 3) or the CTV as PTV (*n* = 2) (refer to Table 3).

**Table 3:** Planning Parameters for included studies.

Author	Prescription	Energy	Tesla	Treatment planning system	Gross tumour volume	Clinical tumour volume	Planning target volume	Beam arrangement	Organs at risk
van Heijst 2013^19^	38.5/10, 42.56/16	6 MV	0, 0.35, 1.5 T	Independent TPS	Preoperative volume or postoperative seroma + surgical clips	GTV + 15 mm	CTV + 5 mm (excluding skin)	7 field IMRT	Heart, lungs, contralateral breast, body (comprising of all unspecified tissue) & skin 5 mm
Esmaeeli 2014^20^	50/25	6 MV	0, 0.25, 1.5	Geant4 Monte Carlo code	–	Lateral border of sternum + midaxillary line	CTV + 5 mm	Tangents	Left lung, heart, chest wall, skin
Kim 2015^21^	38.5/10	Co60	0, 0.35	ViewRay TPS	Lumpectomy cavity	–	GTV + 10–20 mm	6–15 field IMRT	Ipsilateral lung, contralateral lung, heart, inner 3 mm & outer 3 mm skin
Mahdavi 2015^22^	50/25	–	0.5, 1.5	CorePlan (v3.5.0.5)	–	Whole breast	CTV + 5 mm	Tangents	Ipsilateral lung, contralateral lung, heart, inner 3 mm & outer 3 mm skin
Acharya 2016^12^	38.5 Gy/10	Co60	0.35	ViewRay TPS	Surgical cavity	Surgical cavity + 10 mm	CTV = PTV	–	Uninvolved normal breast, contralateral breast, ipsilateral lung, contralateral lung, heart
Chen 2016^23^	45 Gy	6 MV	0, 1.5	Monaco (v5.09.07)	–	–	Whole involved breast	Tangents	Heart, contralateral lung, ipsilateral lung, contralateral breast, skin 5 mm
Fischer‐Valuk 2017^6^	–	Co60	0.35	ViewRay TPS	Surgical cavity	–	PTV = GTV + 10 mm margin (excluding CW, pectoral muscles & 5 mm from skin)	–	–
Kim 2017^24^	40/5	7 MV	0, 1.5	Monaco (v.5.09.07)	Surgical cavity	–	GTV + 10 mm	Tangents 5 field IMRT VMAT	Heart, lung, skin 3 mm, skin 5 mm
Jeon 2017^21^	38.5/10	Co60	0.35	ViewRay TPS	Seroma	GTV + unequal expansion (10–15 mm)	CTV = PTV	–	–
Henke 2018^6^	40/5	Co60	0.35	Independent TPS	Lumpectomy cavity	–	GTV + 10 mm		–
Park 2018^22^	38.5/10	Co60	0.35	ViewRay TPS	Lumpectomy cavity	–	CTV + 10–20 mm	6 field IMRT	Uninvolved normal breast, contralateral breast, ipsilateral lung, contralateral lung, heart, skin
Charaghvandi 2019^23^	20/1, 18/1, 15/1	7 MV	1.5	Monaco (v 5.19.01)	Gross tumour	GTV + 20 mm (excl. CW & first 5 mm of body surface)	CTV = PTV	7 field IMRT	Heart, ipsilateral lungs, contralateral breast, chest wall, skin 5 mm
An 2019^24^	3/1	Co60	0.35	ViewRay TPS	–	–	–	–	–
Nachbar 2020^27^	40.05/15	7 MV	1.5	Monaco (V. 5.19.03)	–	Surgical clip, seroma/tumour bed including visible postoperative changes	CTV + 10 mm (5 mm from skin surface & limited by 7 mm posteriorly)	7 field IMRT	Ipsilateral and contralateral breast, heart, ventricles, left and right coronary artery, lungs, skin 5 mm, lung 5 mm
Groot Koerkamp 2020^29^		Co60, 7 MV	0.35,1.5	–	–	–	–	–	–
Mönnich 2020^30^		7 MV	1.5	Monaco (V 5.4)	–	–	–	–	–

0 T, no magnetic field; Co60, Cobalt 60; CTV, clinical tumour volume; CW, chest wall; GTV, gross tumour volume; Gy, grey; mm, millimetre; MV, megavoltage; PTV, planning tumour volume; T, tesla; TPS, treatment planning system; V, version.

Treatment planning systems included ViewRay (MRIdian) (*n* = 6), followed by Monaco (Elekta) (*n* = 5). Other systems were independent (Geant MC and Coreplan). The MRIgRT systems were cobalt 60‐based (*n* = 7) and LINAC based (*n* = 6), with beam energy of 6 MV (*n* = 4) or 7 MV (*n* = 3). Magnetic field strengths included 0 T (no magnetic field present) to 1.5 T (refer to Table 3). Particular angles on the ViewRay system (20–22°) and Elekta (8–18°) cannot be used due to the cryostat pipe. In addition, 130–150° and 210–230° on the Elekta system, are to be avoided due to high density material in the treatment couch causing unwanted dose effects during daily plan adaptation.^29^

Geometric image distortion was discussed in two studies^6^, ^29^ and is an important consideration when deciding PTV margins and assessing dosimetry. Distortion may arise from system related factors (i.e. magnetic field inhomogeneities or gradient non‐linearity), specific scanner characteristics or sequencing parameters. Increasing the distance between the target volume and the MR‐linac isocentre can lead to system related distortions due to gradient non‐linearity. For the Elekta MR‐linac (1.5 T), maximum displacements of 2.0 mm were found within 17.5 cm from the isocentre. For the ViewRay ^60^Co‐system (0.35 T), this was 1.9 mm, but larger distortions were observed further from the central axis. To account for this inherent issue, it is advised to include this margin in the PTV. Distortion caused by the patient is particularly evident at the tissue‐air interface, with mean maximum distortions at 3.0 T having been found to increase from 1.4 to 3.7 mm in a phantom to 2.7–11.3 mm in patients (including setup uncertainties).^29^

Of the 10 APBI studies, OAR dose assessment was evaluated using constraints from the RAPID trial^32^ and dose volume histograms (DVH). There was no evident or statistically significant effects to the PTV due to the magnet for APBI treatments.^26^ PTV coverage was achievable and feasible with the MR‐linac (Refer to Table 3). Conventional OAR included heart, contralateral and ipsilateral lung. Due to the presence of a magnet, skin dose and chest wall dose were evaluated. Skin was defined as the first 5 mm under the patient external contour (*n* = 5), or 3 mm deep (*n* = 2), with one study using both (Table 4).

**Table 4:** Comparison of OAR and PTV dose levels in APBI studies that reported values.

Target/OAR	Elekta Unity			MRIdian	
	0	0.35	1.5	0	0.35
PTV					
D90%	97.0^19^	97.0^19^	97.0^19^		
	99.5^27^	99.9^27^			
D95%	45.0^24^			98.1^21^	98.9^21^
		99.3(S)^26^ 99.7(P)^26^			
D107%	0^19^	0^19^	0^19^	39.0 ^21^	39.7^21^
	2.1^24^		0^24^		
Lung					
MLD (Gy)	2.1^19^	2.0^19^	1.8^19^		
				7.7^21^	7.7^21^
	2.6^24^		2.9^24^		
			0.9(S)^26^ 0.4(P)^26^		
			3.7^27^		
V5 Gy (%)	25.1^19^	23.1^19^	20.3^19^		
V20 Gy (%)	2.3^19^	2.3^19^	1.9^19^		
Heart					
Dmean	4.3^24^		4.6^24^	4.6^21^	4.7^21^
			0.8(S)^26^ 0.8(P)^26^		
			1.0^25^		
D2cc (Gy)	6.9^19^	8.0^19^	0.4^19^		
V5 Gy (%)	8.0^19^	6.2^19^	6.0^19^		
V10 Gy (%)	0.4^19^	0^19^	0^19^		
Skin					
D2cc (Gy)	35.5^19^	35.2^19^	35.6^19^		
	39.7^27^		40^27^		
D1cc (Gy)			14.7(S)^26^ 15.0(S)^26^		
Dmax (Gy)	45.4^24^		41.3^24^	32.5^21^	37.5^21^
	28.0^27^		31.5^27^		
Dmean (Gy)	5.2^19^	5.6^19^	5.8^19^		
Chest wall					
D20cc (Gy)			12.4(S)^26^ 4.3(P)^26^		
	39.7^27^		37.6^27^		

P, prone; S, supine.

### Organ at risk dose reporting

Dose to all OAR was not statistically significant compared to conventional treatment.

### Skin

In the presence of a 1.5 T magnet, the mean skin dose reported for tangents was 33.2Gy, whole breast irradiation (7 field) 29.8 Gy and APBI (7 field) was 5.8 Gy.^19^ Skin dose outcomes were dependent on the type of plan delivered (i.e. tangential, IMRT, VMAT). Dose to the skin was elevated with WBI in the presence of the magnetic field; however, for the APBI technique, skin dose was negligible (Table 2). Progressing from IMRT angles to VMAT significantly reduced the D1cc and V30 skin dose by 8% and 28%, respectively.^21^

### Lung

Mean lung dose remained low in APBI treatment (approximately 2 Gy). There were no statistical differences in V5 and V20 in the presence of the magnet. A reduction in mean lung dose of 0.9–0.4 Gy was seen in a trial comparing the supine and prone position.^26^

### Chest wall

The chest wall D20cc was 4.3 Gy in the prone position compared to 12.4 Gy in the supine position.^26^

### Heart

Heart dose was not statistically affected by the presence of a magnet. With no magnet present, the D2cc for heart was 6.9 Gy compared to 6.2 Gy for 0.35 T. No significant difference was observed between varying magnet strengths to the heart (i.e. 6.2 Gy for 0.35 T compared to 5.8 Gy for 1.5 T).^26^

### Electron return effect/electron stream effect

The potential implications of secondary electrons within the magnetic field were discussed in 14 of the included studies, highlighting the importance of these interactions for breast MRIgRT (Table 4). The electron return effect (ERE, *n* = 7) and electron stream effect (ESE, *n* = 5) effect were evaluated. There were no reports of the ERE from ViewRay MR‐IGRT. Potential solutions to avoid unwanted elevated dose at the skin or other high‐to‐low density interfaces caused by ERE are to increase number of IMRT fields (*n* = 3), and include the effects on tissue in the plan optimisation (*n* = 4). To reduce the unwanted irradiation of normal tissue resulting from the ESE, it was suggested to plan with multiple IMRT beam directions (*n* = 2) and with bolus on the patient (*n* = 3). Bolus was applied to the patient in three studies; to the chin,^27^ chest wall^20^ and jaw, shoulder and arm.^27^, ^28^

### Quality assurance

Quality assurance (QA) was addressed by one paper using an octagonal phantom and detector array. Plans were measured with phantom orientations optimised for specific beam gantry angles. This method was proven suitable for APBI treatment plans, which can often be off axis if target volumes are lateral.^30^

### Treatment

Treatment techniques of the included studies were tangential beams (*n* = 4), 5–15 field IMRT (*n* = 6) and VMAT (*n* = 1). The favoured treatment option was 5–7 field IMRT due to greater dose conformity compared to 3DCRT.^28^, ^31^ Fractionation scheduling was varied between studies (refer to Table 3). A majority of studies used a regime of 38.5 Gy in 10 fractions (*n* = 5). However, single‐dose fractionation (15, 18, 21 Gy) was also assessed^26^ (refer to Table 2).

Patient positioning was outlined in three articles.^6^, ^12^, ^29^ Patients were aligned to CT simulation tattoos. A volumetric MRI image was then acquired and the GTV match confirmed or manually recontoured at the MRIgRT console.^6^ Acharya et al.^12^ utilised rigid anatomical registration with the simulation and daily treatment image. The surgical cavity was visualised and used to verify alignment.^29^

Motion management strategies on the ViewRay MRIgRT system ranged from patient controlled shallow respiration during simulation and treatment,^27^ to cine‐MR assessed cavity motion^6^, ^12^ and gated treatments.^6^, ^9^, ^28^ Adapt to shape^6^, ^25^ and adapt to position^27^ treatments were delivered. Total patient in‐room‐time average ranged from 28 min^6^ to 30.5 min,^32^ respectively. Nachbar et al.’s^27^ study was the only paper to outline a breakdown of specific workflow times.

Varying forms of ART included; (1) assessing/tracking cavity motion,^12^ (2) gating treatment^6^, ^9^ and (3) adapting to shape for that days anatomy.^27^ Online ATS was conducted by the multidisciplinary team and reportedly took approximately 26 min to perform recontouring, reoptimisation and QA.^6^ When treating APBI, the overall motion of the tumour cavity was low (<3 mm) in both anterior–posterior (AP) and superior–inferior (SI) directions.^21^ Similarly, mean AP and SI displacements by Fischer‐Valuk et al.^6^ was 0.6 ± 0.4 and 0.6 ± 0.33 mm. In Jeon et al.’s study, of their 37 patients, 4 (10.8%) experienced seroma increase during the period between CT and 1st fraction and 33 exhibited a decrease SV over their treatment period. It was advised that no patients’ seroma size increased in the last period of their accelerated schedule, suggesting that adaptive planning on the 6th fraction does not threaten coverage.^25^

## Discussion

This scoping review identified 16 primary studies addressing the use of an MRL for breast cancer radiotherapy. The literature verifies that APBI is the most commonly used technique due to the superior soft tissue delineation, making volume definition more straight forward compared to CBCT. Moreover, treatment can be adapted online, based on daily anatomy imaged by MR and intrafractional imaging to ensure precise delivery.^27^ PTV coverage was achievable and comparable to APBI treatment on a conventional linac. Overall dose to OAR was not statistically significant compared to conventional treatment. In fact, lower doses were routinely seen for OAR since overall prescription is reduced compared to standard fractionation.

Considerations including magnet strength, beam energy, source‐to‐axis and field size are essential in MR planning.^19^, ^29^ Due to magnetic field presence, geometric distortion requires attention. Potential inaccuracy in the assignment of air and tissue electron density may arise. Consequently, inaccurate dose calculations may occur. To reduce the effects, the target volume should be positioned as close to the scanner isocentre as possible, which can be challenging for lateral breast tumours. To rectify this, the patient can be offset on the scanner towards the contralateral breast with the treated breast closer to the isocentre. Limiting factors to this solution is the space inside the bore. Software can also be used to reduce gradient non‐linearity. A lower field strength or high receiver bandwidth can help to reduce magnetic field inhomogeneity and patient induced susceptibility, however, reducing signal‐to‐noise ratio.^6^, ^29^

The planning MR and CT are overlayed via rigid or transformable methods.^29^ The RO utilises the MR image to determine tumour/ bed location in the breast. It is recommended that the GTV encompass the tumour bed including visible seroma/surgical clips, preoperative tumour location whilst also taking into account the microscopic tumour free margins.^29^ CTV/PTV contouring differs between many studies (refer to Table 2). The prone position was found to elongate the tumour bed. Consequently, the mean CTV and PTV volumes were significantly higher for patients in this position compared to supine.^26^

A novel challenge presented with the MRL linac was ESE and ERE. Both circumstances are generated by the presence of the magnetic field causing the Lorentz force. However, their effect on treatment differs. ESE results in undesired irradiation outside the treatment field, whilst ERE results in increased dose deposition at tissue‐air interface. Factors contributing to increase ERE can include oblique treatment angles, magnetic field strength, electron energy and relative density differences at the tissue interface.^30^ It is strongly advisable to have skin as an OAR constraint in planning to regulate the amount of dose received (refer to Table 5).

**Table 5:** Potential solutions for ERE and ESE.

Effect	Configuration	Potential solution
ERE	Tangential field WBI 2 field IMRT	Increase number of IMRT fields, for example, 7‐beam APBI approach (IMRT)^19^
	Tangential 2–3 beams HPBI	IMRT planning IMRT with increased beam angles or a VMAT configuration Inverse planning that includes magnetic field^24^
	IMRT and VMAT plans in 1.5 T transverse magnetic field	Plan reoptimisation to include transverse magnetic field Multiple beam directions in IMRT/VMAT plans ^23^
	Tangential fields with transverse magnetic field 0.25–1.5 T	Reversible magnetic field with direction of magnetic field cranial‐caudal for medial and vice‐versa for lateral beam, at lower magnetic field of 0.25 T^20^
	Tangential fields with LRBP and TRBP geometries 0.5–1.5 T	Both geometries exhibit dose reduction to lung, heart, contralateral organs breast and chest wall skin Improved dose homogeneity for PTV (sharper edge DVH curves) for higher magnetic field of 1.5 T in TRBP^22^
	PBI 7‐beam IMRT 1.5 T	During RT planning Display and assess low value isodose lines Delineate skin as OAR Optimise the plan according to a higher dose to skin and/or air‐tissue interfaces^27^
ESE	Target volumes located close to or including surface	Shielding with ≥1 cm bolus during treatment^24^ Reducing projected area of cross‐section of treatment beam on irradiated surface (beam angle, field size, treatment distance, that is, SSD)^31^
	APBI with target volumes depth of 5 mm and tumour located in region of upper breast	Treatment with 1 cm bolus on patient jaw, ipsilateral shoulder and arm^28^
	PBI with static IMRT	Multiple IMRT beam directions^21^
	PBI 7‐beam IMRT 1.5 T	During RT planning Simulation scan of patient up to the nose Display and assess low value isodose lines Delineate skin as OAR Optimise the plan according to a higher dose to skin and/or air‐tissue interface Treatment with bolus on chin^27^

Skin‐dose outcomes were dependent on magnet strength. In the presence of a magnet, dose to skin in tangential fields is increased. Dose to the skin was elevated with WBI in the presence of the magnetic field, but in APBI the effects were negligible (Table 4) Nachbar et al.^27^ illustrated that going from a normal linac to an MRL (i.e. 0–1.5 T) will lead to an increase in dose to the skin; 28.0–31.5 Gy, respectively. Van Heijst et al.^19^ saw a similar trend; 27.9 and 29.8 Gy. ERE was greater in higher magnetic fields, leading to higher skin doses. Dosimetric effects on skin observed at 0.35 and 1.5 T was seen with significant increases in skin dose with magnetic field strength with conventional treatment by tangential field WBI compared to APBI. However, a limitation to these findings was that the fractionation between the two groups was different.

Skin dose was also dependent on the type of plan delivered (i.e. tangential, IMRT, VMAT). Increasing the number of beam angles (i.e. utilising VMAT as an alternative to IMRT) with the magnetic field on reduces skin dose.^31^ This effect occurs due to increasing numbers of beam angles having a lesser impact at the entry points compared to the beam exit points.^24^ Care should be taken when interpreting these results as there was only one study that included VMAT planning. In addition, VMAT is not currently deliverable on the MR‐linac, therefore, evidence suggests IMRT with approximately 6–7 beams is the best beam arrangement for APBI.

Other factors influencing dose to the skin and CW include the depth and location of the tumour. ESE was seen to increase when the tumour was located in the region of the upper breast. On the contrary, no ESE was observed when the tumour was located in the region of lower breast. The effects of ESE in skin and CW can be mitigated by using bolus. CW dose was seen to increase in all patients at 1.5T compared to conventional treatment.^20^ The use of bolus enables the high dose to be shifted from CW and create a uniform dose in the breast tissue.^20^ Both the effects require consideration in planning processes and compensation if possible. More recent retrospective papers have found that ESE/ERE was not associated with increased risk of acute toxicities.^27^

Contralateral lung dose was not statistically significant with the presence of a magnet across the investigated range of magnetic field strengths.^26^ The prone position was shown to be beneficial in minimising dose to the ipsilateral lung and chest wall. Therefore, depending on available equipment, prone may be a beneficial setup position to consider. The differences between VMAT/IMRT were not statistically significant for heart dose making both treatment modalities viable options.^26^

Online adaptation and the monitoring of seroma/tumour motion is of immeasurable value. However, more data is required for margin sizes and gating windows as this remains an inconsistency between centres. A recommendation of standardisation between centres with the same MR‐linac vendor could be of use. Groot Koerkamp et al.^29^ suggested individualising PTV margins based on individual patients’ cine‐MR data from simulation. An additional factor to consider is the trade‐off between treatment time and plan quality. The benefits of this function are monumental in breast treatment delivery; however, a balance is required. Another impression by the authors of this review is that more studies are required to obtain qualitative and quantitative details on patient experience and if longer lasting implications exist.

Our scoping review has some limitations. It is critical to note that the included studies are not exhaustive due to the novelty of this machine. Discussions headings were based on the diversity of reported information in the included journal articles. Authors were hopeful to extract information regarding quality assurance processes, MR safety, tolerability, sequencing for imaging and image quality, planning and MR simulation, however, such information was briefly covered or not included in the evaluated studies. This is likely due to our specific inclusion criteria where papers, which discussed radiotherapy, MR‐linac and breast cancer were included. A broader criteria including breast MR imaging specific papers would have provided the ability to extract more information on these excluded areas. In the future, broadening the inclusion criteria to gain more insight would be valuable. It has become apparent during the installation of the MR‐linac at our department that many other departments and our own will employ an MR radiographer to assist the development of imaging, MR safety, etc. protocols.

## Conclusion

In conclusion, the current available data suggest the MR‐linac will is most commonly indicated for APBI treatment due to excellent soft tissue visualisation. This treatment will be suitable for patients who have low stage breast cancer and will minimise the irradiation of healthy breast tissue. The authors recommend the conduct of a future systematic review.

## Funding Information

This research received no specific grant from any funding agency in the public, commercial or not‐for‐profit sectors.

## Conflict of Interest

The authors declare that there is no conflict of interest.
